# Laparoscopic Excision of Douglas Pouch Fibroma in Contact With the Sigmoid Colon

**DOI:** 10.7759/cureus.78930

**Published:** 2025-02-13

**Authors:** Angelos Daniilidis, Tilemachos Karalis, Evangelia Mareti, Konstantinos Nikolettos, Fani Gkrozou

**Affiliations:** 1 1st Department of Obstetrics and Gynecology, Papageorgiou General Hospital of Thessaloniki, Thessaloniki, GRC; 2 Department of Obstetrics and Gynecology, Genesis Hospital, Thessaloniki, GRC; 3 Department of Obstetrics and Gynecology, University Hospital of Alexandroupolis, Alexandroupoli, GRC; 4 Department of Obstetrics and Gynecology, University Hospital of Ioannina, Ioannina, GRC

**Keywords:** douglas pouch, laparoscopy, parasitic fibroid, remnant fibroid, sigmoid colon, uterine fibroid

## Abstract

Pelvic tumors, especially those in difficult locations such as tumors in the rectouterine pouch in contact with the sigmoid colon, pose therapeutic approach difficulties due to the increased risk of complications, such as intestinal perforation. In practice, open surgical technique is often used even though laparoscopy has more advantages, due to lack of familiarity with delicate endoscopic maneuvers. This article describes the laparoscopic excision of a pelvic mass (residual fibroid after a previous myomectomy) implanted in the rectouterine pouch and in contact with the sigmoid colon. The patient is a 49-year-old woman with a history of laparoscopic excision of a 10 cm FIGO 4 (International Federation of Gynecology and Obstetrics Stage 4) fibroid three years ago. She presented to the outpatient clinic for prenatal care initiation, where the ultrasound revealed a solid mass in the pelvis measuring 6x6 cm. The MRI differential diagnosis at 28 weeks of gestation included sarcoma, ovarian cancer, and fibroid. Cancer markers were negative. Conservative management was decided until delivery. During pregnancy, the mass remained stable in size and morphology. At 39 weeks, a selective cesarean section was performed due to a history of myomectomy, during which the mass was identified between the intestinal loops. The ultrasound three months postpartum depicted the mass measuring 4.5x4.5 cm located in the rectouterine pouch. A laparoscopic approach was decided, during which the mass was initially identified and then dissected from the surrounding tissue using bipolar bovie and laparoscopic scissors. Delicate manipulation was needed when dissecting in order to avoid bowel perforation. The mass was removed from the peritoneal cavity using an endoscopic bag to prevent tissue dissemination. The histological diagnosis was fibroid. This report demonstrates that pelvic tumors in difficult locations like in contact with the sigmoid colon can be safely removed by an experienced laparoscopic surgical team.

## Introduction

Gynecological laparoscopy stands as a pivotal advancement in modern surgical techniques, revolutionizing the management of difficult pelvic mass extraction surgeries. The minimally invasive procedure offers remarkable precision and efficacy in diagnosing and treating various gynecological conditions, including ovarian cysts, endometriosis, and fibroids [1]. The significance of gynecological laparoscopy lies not only in its ability to minimize surgical trauma and accelerate recovery but also in its capacity to navigate complex anatomical structures with enhanced dexterity, thereby optimizing outcomes for patients facing challenging pelvic masses. The unparalleled access to the pelvic region that laparoscopic techniques provide allows for meticulous examination and targeted intervention, with the sole restriction being the surgeon’s laparoscopic skills [2].

The occurrence of retained myomas following myomectomy is exceedingly rare, but it poses unique challenges when discovered in unexpected clinical settings, such as pregnancy. Myomectomy is typically a definitive treatment for uterine fibroids, aiming to relieve symptoms and improve fertility outcomes. However, complications such as retained or "lost" myomas are occasionally reported, especially in cases involving laparoscopic morcellation, where fragments might unintentionally remain in the peritoneal cavity that implants and grow within the abdomen after morcellation forming parasitic myomas [3]. This case report presents a unique instance of a retained myoma diagnosed during pregnancy monitoring that was successfully treated laparoscopically postpartum, underscoring the importance of thorough intraoperative evaluation and vigilance in postoperative follow-up to prevent such outcomes.

## Case presentation

**Figure 1:**
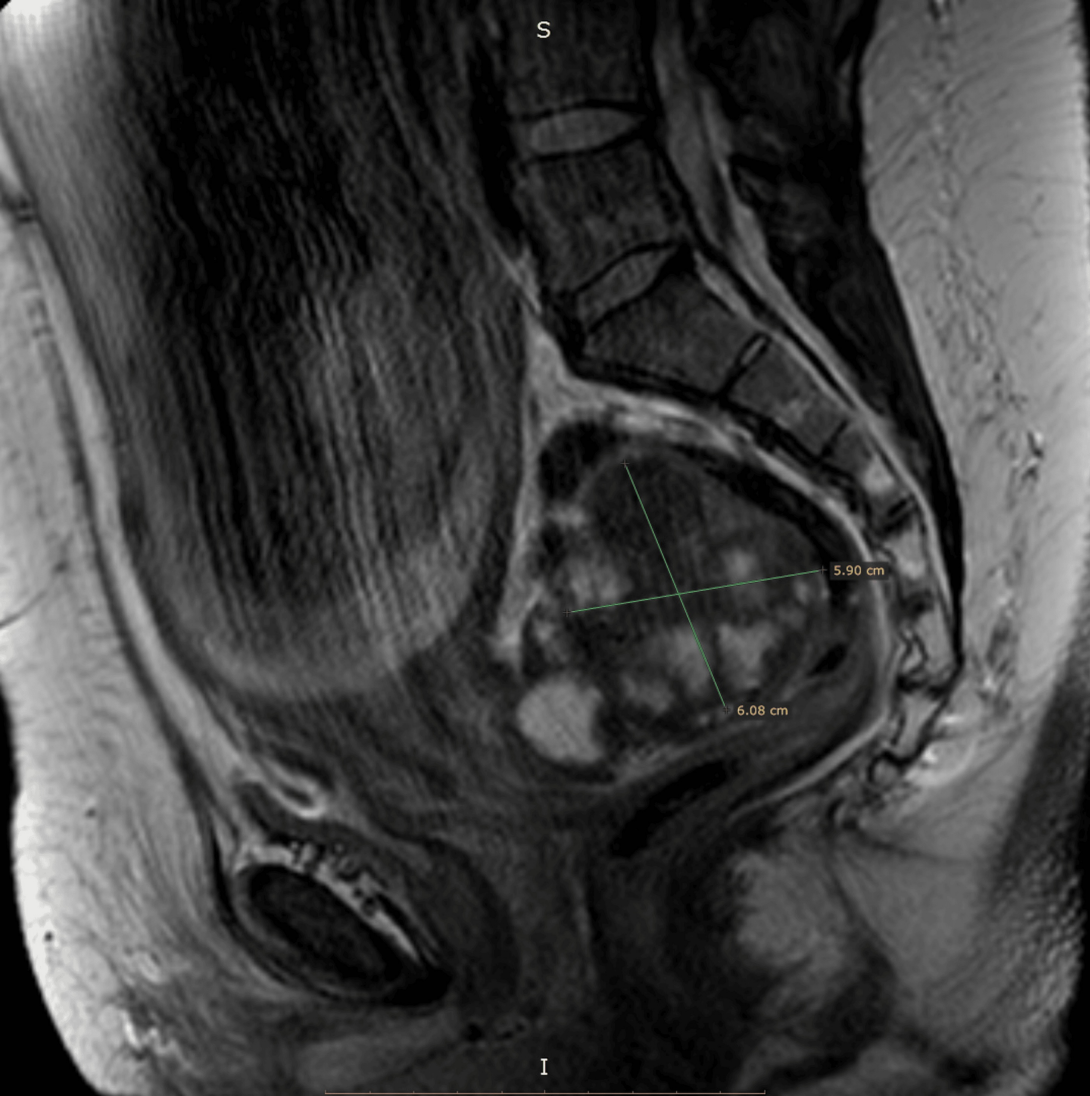
MRI at 28 weeks of gestation, dimensions of the tumor and location (sagittal plane) MRI, magnetic resonance imaging.

**Figure 2:**
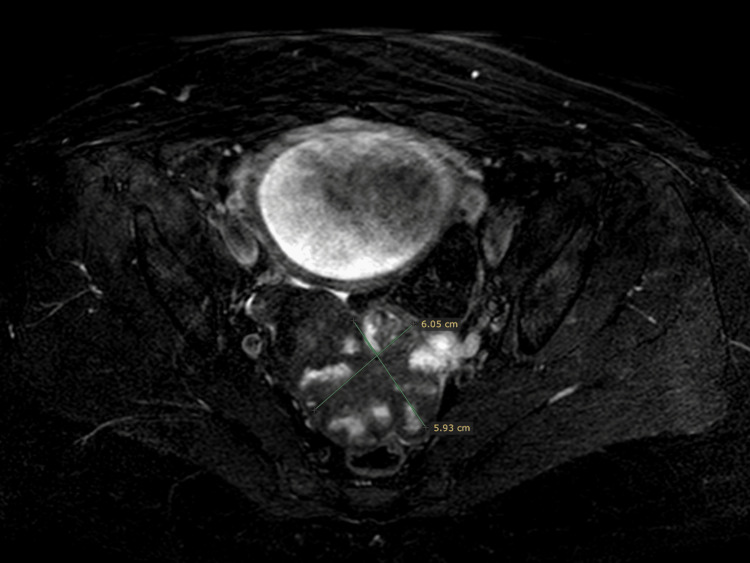
MRI at 28 weeks of gestation, dimensions of the tumor and location (axial plane) MRI, magnetic resonance imaging.

The patient is a 49-year-old woman, with a history of laparoscopic excision of a 10 cm FIGO 4 (International Federation of Gynecology and Obstetrics Stage 4) fibroma three years ago. She presented to our outpatient office for routine pregnancy monitoring, where an ultrasound demonstrated a 6x6 cm cauliflower-like pelvic tumor. A magnetic resonance imaging (MRI) was performed at 28 weeks of gestation, to better visualize the tumor and its relation with the surrounding structures (Figures 1, 2). 

Differential diagnoses of the MRI included sarcoma, ovarian tumor, and fibroma. Tumor markers were negative. Expecting management was decided until labor, and during pregnancy, the tumor remained stable in size and morphology. At 39 weeks, elective cesarean section was performed due to the history of fibromyomectomy, during which the tumor was recognized among the intestinal loops. Ultrasound imaging three months postnatally depicted the 4.5x4.5 cm tumor located on the Douglas pouch.

The laparoscopic approach was chosen for the excision of the pelvic mass. After entering the abdominal cavity, we visualized the pelvis where we recognized a cauliflower-like pelvic tumor in the Douglas pouch. The tumor had a multinodular appearance and was in close proximity to the sigmoid colon. After distinguishing the margins of the mass, we began separating it from the surrounding healthy tissue, using laparoscopic scissors, bipolar bovie, and the suction/irrigation cannula. With the bipolar bovie, we coagulated some spots peripherally at the margins between the tumor’s serosa and the pelvic peritoneum in order to avoid bleeding and to provide openings for the instruments to dissect. Using laparoscopic scissors, we cut the overlying serosa and peritoneum, and the underlying tissue in order to get to the masse’s stem. The tumor was very mobile, sweeping along the underlying sigmoid when moved. Special attention was needed not to traumatize the surrounding tissue, as underneath it was the sigmoid colon and there was a high risk of bowel perforation. In the end, the tumor was fully excised and placed inside a specimen retrieval bag in order to extract it from the abdominal cavity avoiding tissue dispersion. Finally, the traumatic surface of the pelvic peritoneum is coagulated carefully and properly to avoid any bleeding. The following video contains the highlights of the forementioned procedure (Video 1). 

**Video 1:** Laparoscopic dissection and extraction of the pelvic mass (procedure highlights)

**Figure 3:**
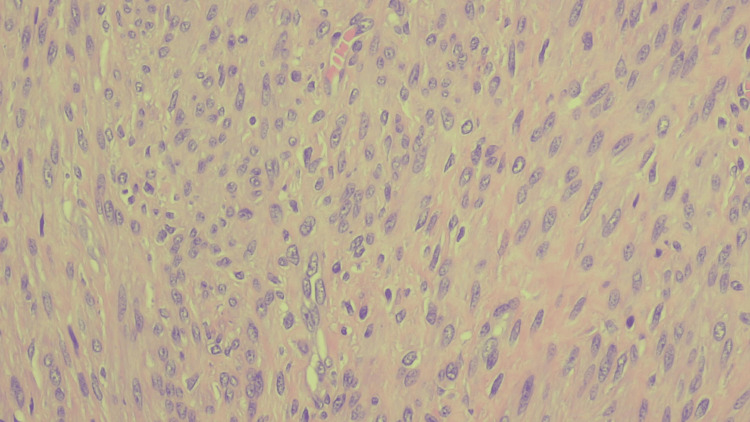
Microscopic image of the fibroid with benign histologic features, moderate cellularity, and absence of atypia, mitoses, or necrosis

The histopathology report provided the diagnosis of fibroid with bland histological features: moderate cellularity, and no atypia, mitoses, or necrosis (Figure 3).

The postoperative period was without complications. The patient was discharged the third day after surgery, and a postoperative follow-up of two appointments was arranged, in 10 days and one month after discharge. In both the follow-up appointments, the patient was asymptomatic, and underwent clinical and ultrasonographic examination, with no evidence of complications.

## Discussion

The case presented here highlights the successful use of laparoscopic techniques in the excision of a challenging pelvic mass located in the rectouterine pouch. The utilization of laparoscopic instruments allowed for precise dissection and removal of the fibroid, which was intricately positioned in close proximity to the sigmoid colon. The meticulous approach taken by the surgical team in identifying the margins of the mass and utilizing appropriate tools contributed to the successful outcome of the procedure.

Furthermore, the management of the tumor during the patient's pregnancy and the decision for an elective cesarean section took into consideration the patient's history of fibromyomectomy, demonstrating a multidisciplinary approach to her care. The stability of the tumor during pregnancy and its subsequent growth postnatally underscore the importance of close monitoring and timely intervention.

A thorough literature search (PubMed, Cochrane, and Google Scholar databases) resulted in very few reports of similar cases. Van der Meulen et al. conducted the most recent systematic review on parasitic myomas, focusing on studies where the use of laparoscopic morcellation sends tissue fragments of the tumor to various places in the endometrial cavity [4]. The review included 44 studies with a total of 69 women and concluded that the median time between diagnosis and surgery was 48 months (1 to 192 months), the mean number of parasitic myomas was 2.9 (ranged from 1 to 16), and the overall incidence of parasitic myomas after laparoscopic morcellation was 0.12-0.95%. After this systematic review, only two cases were published. The first one was a case of multiple parasitic myomas located at the pelvis and anterior abdominal wall two years after laparoscopic myomectomy, which were removed laparoscopically [5]. The second was a case of a woman with a parasitic myoma at the right anterior abdominal wall, one year after transabdominal myomectomy, without the use of a morcellator, that was laparoscopically removed [6]. To our knowledge, no other case was published with the presence of a retained myoma during pregnancy, along with its challenges regarding both the diagnosis and the management of the condition.

This case emphasizes the significance of gynecological laparoscopy in addressing complex pelvic masses, offering a minimally invasive yet highly effective alternative to traditional open surgery. The ability to navigate intricate anatomical structures while minimizing trauma to surrounding tissues, as demonstrated in this case, showcases the benefits of laparoscopic techniques in optimizing patient outcomes.

The successful excision of the pelvic mass in this case serves as a testament to the advancements in modern surgical approaches, underscoring the continued evolution and refinement of laparoscopic procedures in gynecological surgery.

## Conclusions

Overall, this case demonstrates the continued evolution and refinement of laparoscopic procedures, reaffirming the pivotal role of gynecological laparoscopy in optimizing patient outcomes and revolutionizing the management of challenging pelvic masses. The complexity of such surgeries requires not only technical proficiency but also a comprehensive understanding of pelvic anatomy and meticulous surgical planning. With an experienced laparoscopic team, the excision of difficultly located masses laparoscopically is a safe and efficient method that needs to be examined thoroughly before trying other, more invasive techniques.
